# Paraptosis Cell Death Induction by the Thiamine Analog Benfotiamine in Leukemia Cells

**DOI:** 10.1371/journal.pone.0120709

**Published:** 2015-04-07

**Authors:** Naomi Sugimori, J. Luis Espinoza, Ly Quoc Trung, Akiyoshi Takami, Yukio Kondo, Dao Thi An, Motoko Sasaki, Tomohiko Wakayama, Shinji Nakao

**Affiliations:** 1 Department of Hematology Oncology, Kanazawa University Hospital, Kanazawa University, Kanazawa, Japan Takaramachi 13–1, Kanazawa, Japan; 2 Department of Hematology Oncology, Aichi Medical University School of Medicine, Nagakute, Japan; 3 Department of Pathology, Kanazawa University, Kanazawa, Japan; 4 Department of Anatomy and Histology, Kanazawa University, Kanazawa, Japan; Queen's University Belfast, UNITED KINGDOM

## Abstract

Benfotiamine is a synthetic thiamine analogue that stimulates transketolase, a cellular enzyme essential for glucose metabolism. Currently, benfotiamine is used to treat diabetic neuropathy. We recently reported that oral benfotiamine induced a temporary but remarkable recovery from acute myeloid leukemia in an elderly patient who was ineligible for standard chemotherapy due to dementia and renal failure. In the present study we present evidences that benfotiamine possess antitumor activity against leukemia cells. In a panel of nine myeloid leukemia cell lines benfotiamine impaired the viability of HL-60, NB4, K562 and KG1 cells and also inhibited the growing of primary leukemic blasts. The antitumor activity of benfotiamine is not mediated by apoptosis, necrosis or autophagy, but rather occurs though paraptosis cell death induction. Mechanistic studies revealed that benfotiamine inhibited the activity of constitutively active ERK1/2 and concomitantly increased the phosphorylation of JNK1/2 kinase in leukemic cells. In addition, benfotiamine induced the down regulation of the cell cycle regulator CDK3 which resulted in G1 cell cycle arrest in the sensitive leukemic cells. Moreover, combination index studies showed that benfotiamine enhanced the antiproliferative activities of cytarabine against leukemia cells. These findings suggest that benfotiamine has antitumor therapeutic potential.

## Introduction

Acute myeloid leukemia (AML) is a rapidly progressing, heterogeneous clonal disorder of hematopoietic progenitor cells characterized by an abnormal expansion of hematopoietic precursor cells with limited or abnormal differentiation that results in the accumulation of immature leukemic blasts. At the molecular level, alterations in the activity of transcription factors controlling hematopoietic differentiation and the deregulated activation of receptor tyrosine kinase signaling pathways constitute the two major genetic events involved in leukemic transformation [1].

Significant progress in understanding the molecular pathogenesis of AML has led to the development of new targeted and chemotherapeutic agents, which has improved the outcomes of patients with AML [2]. However, disease relapse and complications associated with standard chemotherapy present difficult challenges [2,3]. Elderly patients with AML are more susceptible to chemotherapy-related complications. Such patients are often ineligible for intensive chemotherapy and thus are managed solely with conservative approaches [3–5]. Therefore, finding novel therapeutic agents with lower levels of cytotoxicity is necessary.

Benfotiamine (S-benzoylthiamine O-monophosphate), is a water-insoluble synthetic thiamine derivative with a reported bioavailability five-fold higher than that of water-soluble thiamine [6]. Benfotiamine is currently used to prevent the progression of diabetic complications, such as neuropathy, nephropathy and retinopathy [6,7]. In addition, benfotiamine possesses numerous health-promoting properties, including anti-inflammatory, antioxidant and neural protective activities [6,8–10]. However, to date, no studies have demonstrated the direct antitumor effects of benfotiamine.

We recently reported that in a patient with AML who was ineligible for standard chemotherapy due to his advanced age and because he had dementia, chronic renal disease and angina pectoris, the number of peripheral blasts decreased dramatically after receiving monotherapy with oral benfotiamine that was being given to treat low levels of vitamin B1. In that particular patient, leukemia cells became virtually undetectable by 20 days after the initiation of benfotiamine therapy without causing tumor lysis syndrome (Sugimori 2013: 75^th^ annual meeting JSH, PS-2-35). Although the patient eventually died due to leukemia regrowth, we hypothesized that a relation may exist between benfotiamine intake and the transient leukemia remission observed in that patient. In the present study, we report evidences indicating that benfotiamine may have therapeutic potential against AML. Our mechanistic studies suggest that benfotiamine inhibits leukemic cell growth by triggering paraptosis cell death.

## Materials and Methods

### Cell culture

Molt4, THP1, KG1, HL60, Daudi, Raji, CCRF-CEM, K562, HEL and U937 cells lines were purchased from the Health Science Research Resources Bank (Osaka, Japan). Jurkat cells were purchased from ATCC (Rockville, MD, USA). The myeloid leukemia OUN1 [11], NB4[12] and KH88 [13] cells were kindly provided by Dr. M. Yasukawa of Ehime University (Matsuyama, Japan) and the myelodysplastic syndrome cell line TF-1 [14] was obtained from Dr. S. Ogawa of the University of Tokyo (Tokyo, Japan). The EBV+ lymphoblastoid cell lines LCL-1, LCL-2 and LCL-3 were established in our lab and have been described in a previous study [15]. The TF-1 cells were cultured in Iscove's modified Dulbecco's medium supplemented with 20% FBS and granulocyte/macrophage colony stimulating factors. All other cells were cultured in RPMI-1640 medium supplemented with 10% FBS and 1% penicillin and streptomycin in a 5% CO_2_ atmosphere.

### Primary leukemic cells culture

This study was approved by the Institutional Review Board of the Kanazawa University School of Medical Sciences and was conducted in accordance with the *Helsinki Declaration of 1975*, as revised in 2008. Written informed consent to publish this report was obtained from the patient’s family; a copy of the written consent form is available upon request. Primary leukemia cells used in this study were obtained from a patient with AML (Sugimori 2013: 75^th^ annual meeting JSH, PS-2-35). The samples were acquired either from bone marrow (BM) aspirate collected when the patient was admitted to the hospital (designated hereafter as BM-S-blasts) or from a blood sample collected when the patient’s condition worsened, one week before he died (designated hereafter as PB-R-blasts). Other primary leukemia blasts were obtained from the bone marrow or peripheral blood from six patients with newly diagnosed or relapsed AML. Informed consent was obtained from all patients in accordance with the Declaration of Helsinki. BM and peripheral blood mononuclear cells containing more than 90% leukemic blasts were isolated using gradient centrifugation, aliquoted and cryopreserved until use. The cryopreserved blasts for use in the *in vitro* experiments were more than 90% viable, as demonstrated using trypan blue staining. These cells were cultured in RPMI 1640 medium supplemented with 20% FBS, stem cell factor (100 ng/mL), GM-CSF (20 ng/mL) and 1% penicillin and streptomycin. Benfotiamine (Sigma) was solubilized in 1% carboxymethylcellulose (Sigma) as previously described [16]. Leukemia cells were treated with varying concentrations of benfotiamine prepared from a stock solution of 5 mM. The control cells were treated with the diluents.

Cell viability was assessed using a colorimetric MTT metabolic activity assay according to the manufacturer’s instructions (Roche, Minato-ku, Tokyo, Japan). The absorbance intensity was measured on a MultiSkan microplate reader (Thermo Scientific) at 490 nm with a reference wavelength of 620 nm. All experiments were performed in quadruplicate, and the percentage of cell viability was calculated by the following formula: (cell viability) % = (*OD* of drug-treated cells/*OD* of untreated cells) ×100.

The CompuSyn software program (ComboSyn, Inc.; Paramus, NJ) was used to calculate the combination index (CI) to determine the presence of drug interactions affecting cell proliferation. For this purpose, the inhibition of cell proliferation was determined as described in a previous report [17].

### Cell cycle, apoptosis and autophagy studies

A cell cycle analysis was performed using a Becton Dickinson FACS flow cytometer as previously described [18]. Apoptosis of leukemia cells was assessed based on 7AAD/annexin V staining (Invitrogen, Tokyo, Japan) in cells cultured in the presence or absence of benfotiamine [15] and Giemsa staining was performed [17] as previously described. In some experiments, HL-60 cells were pre-treated with 0.25 μg/ml of either cycloheximide or tetrathylammonium, a big potassium channel (BK) blocker, for 2 hours and then cultured for 12 hours with or without benfotiamine (100 μM) and stained with Giemsa. Autophagy was assessed by Western blotting for LC3 protein or by using the Cyto-ID Autophagy detection kit (Enzo Life Sciences), which uses a green fluorescent probe that reacts with autophagic vacuoles and is detectable by flow cytometry.

### Transmission electron microscopy

HL-60 cells treated with or without benfotiamine were fixed by immersion in 2% paraformaldehyde plus 2.5% glutaraldehyde in 0.1 M phosphate buffer (pH 7.4) for 1 hour at 4°C. After washing in 0.1 M phosphate buffer at 4°C, the cells were postfixed in 1% osmium tetroxide for 1 hour at 4°C. They were then washed repeatedly in distilled water, stained with 1% uranyl acetate for 30 min, dehydrated through graded ethanol series and propylene oxide, and embedded in Glicidether (Selva Feinbiochemica, Heidelberg, Germany). Ultrathin sections were cut and mounted onto copper grids, stained with 1% uranyl acetate for 10 min followed by Reynolds lead citrate for 5 min, and then observed on an H-7650 electron microscope (Hitachi High-Technologies, Tokyo, Japan).

### Western blotting

A Western blot analysis of whole-cell lysates was conducted as previously described [17]. The blots were probed with the following primary antibodies: phosphor-CDK2, total CDK2, CDK3, phosphor-CDC2, total CDC2, phospho-ERK1/2, phosphor-JNK1/2 (all from Cell Signaling), CDK4 (Santa Cruz Biotechnology), total p38, total ERK1/2, total JNK1/2, phospho-p38, total p38 MAPK (all from Promega), anti α-tubulin (Sigma) and anti-LC3 (MBL). Immunoreactive proteins were detected as previously described [17].

### Statistical Analysis

All data are reported as the mean ± S.E. The t-test was used to make comparisons between groups. The statistical significance of multiple comparisons was determined using a one-way analysis of variance. Statistical significance was considered to be present p≤0.05. All statistical analyses were performed using the GraphPad Prism software package (San Diego).

## Results

### Antiproliferative effects of benfotiamine on hematological malignant cells

**Fig 1:**
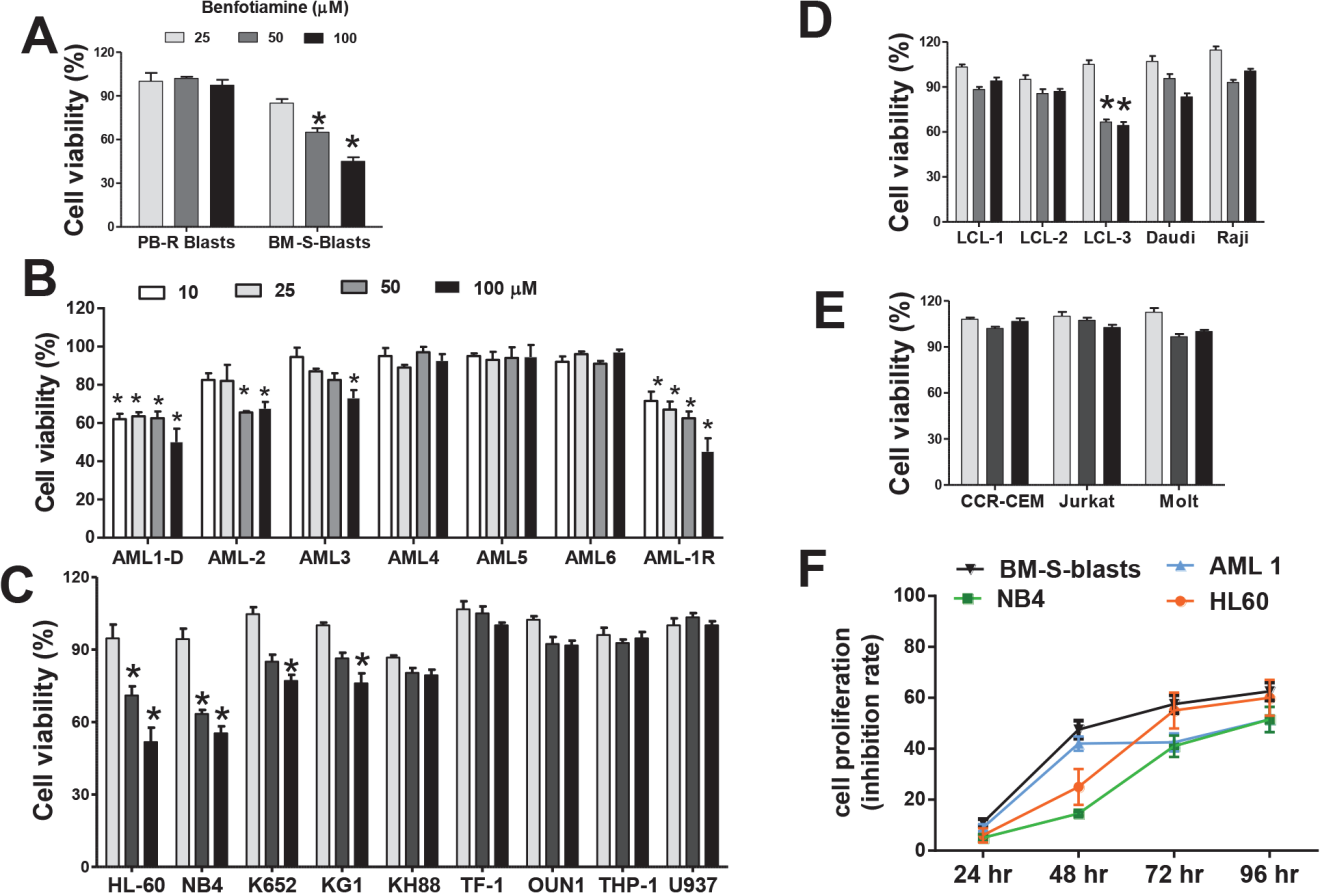
Effects of benfotiamine on the viability of leukemic cells. BM-S and PB-R leukemic blasts (A), primary leukemia cells from six patients with AML (B), myeloid leukemia cell lines (C), malignant B cell lines (D) and T cell leukemia (E) were left untreated or treated with the indicated doses of benfotiamine and cultured for 72 hours. Cell viability was assessed using an MTT assay. The error bars represent relative cell viability with respect to the untreated control cells and are the means (SEM) of three independent experiments. (F) Growth curves of leukemia cells exposed to 50 uM of benfotiamine and analyzed at multiple time points using a colorimetric MTT assay. The average data of three independent experiments are shown.

Because oral benfotiamine induced clinical antitumor responses when given to a patient with AML, who was ineligible for standard chemotherapy (Sugimori 2013: 75^th^ annual meeting JSH, PS-2-35), we performed *in vitro* studies using patient-derived leukemic cells collected at diagnosis (BM-S-blasts) and leukemic cells obtained when the patient’s condition worsened (PB-R-blasts) and determine their viability in the presence or absence of benfotiamine. A colorimetric MTT assay showed that benfotiamine profoundly compromised the viability of the primary BM-S-blasts. Notably, this effect was not observed in the PB-R-blasts (**Fig 1A**) suggesting these cells may have acquired a protective mechanism that enabled them to evade the antitumor activity of benfotiamine.

To further define the antitumor potential of benfotiamine, primary leukemia cells from six AML patients with different cytogenetic characteristics (S1 Table) were cultured in varying concentrations of benfotiamine. Benfotiamine strongly inhibited the growth of leukemia cells obtained, either at diagnosis (AML-1D) or after relapse (AML-1R), from a patient with AML and also showed a moderate inhibitory effect on the leukemia cells from two additional patients (AML-2 and AML-3) (**Fig 1B**). Next, we assessed the effects of benfotiamine on cell viability in 17 tumor cell lines, including Burkitt’s lymphoma (Raji and Daudi), EBV transformed B cells (LCL-1, LCL-2 and LCL-3), T cell leukemia (Jurkat, Molt4 and CCRE-CEM) and myeloid leukemia (KG1, HL-60, K562, U932, KH88, THP-1, NB4, TF-1 and OUN1). We observed a significant decrease in the viability of four out of nine myeloid leukemia cell lines treated with benfotiamine, strong antitumor effects on HL-60 and NB4 cells and slight antitumor effects on KG1 and K562 cells (**Fig 1C**). Significant antitumor effects were also observed in LCL-3 cells and somewhat observed in Daudi cells (**Fig 1D**). Conversely, benfotiamine did not impair the viability in the three T cell leukemia lines tested in this study (**Fig 1E**). To further characterize the antitumor effects of benfotiamine, we assessed the proliferation of responsive cells at several time points following benfotiamine treatment. The results revealed that while the most robust anti-proliferative effects of benfotiamine in leukemia cell lines were observed by 72 hours after treatment, in the primary blasts these effects were detectable 48 hours after exposure to the drug (**Fig 1F**)

### Effects of benfotiamine on cell death mechanisms

**Fig 2:**
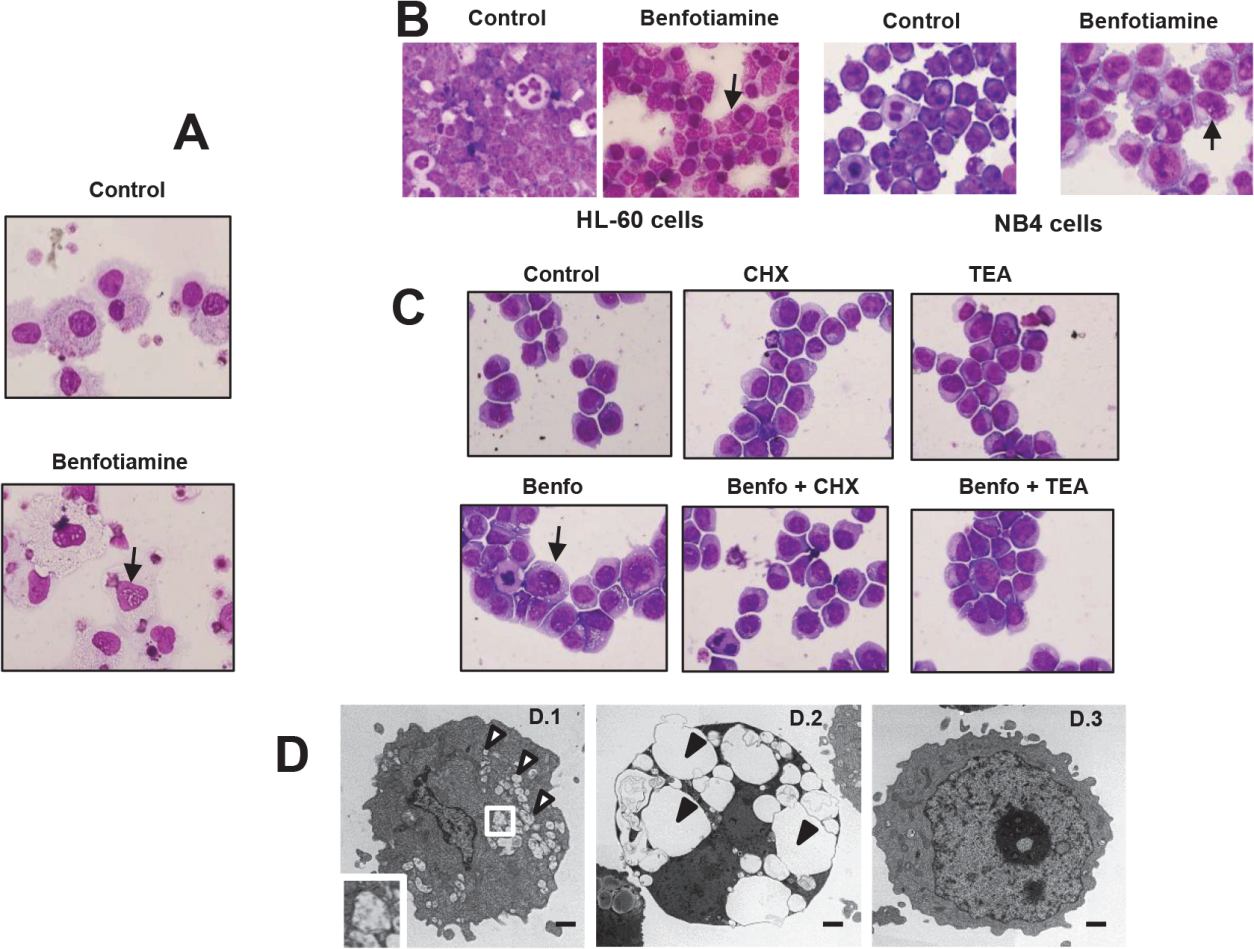
Benfotiamine induced cytoplasmic vacuolization in myeloid leukemic cells. Primary leukemia cells BM-S-blasts (A) were cultured for 72 hours in the presence or absence of benfotiamine (100 μM) and subjected to Giemsa staining. Representative results of three independent experiments are shown. (B) HL-60 and NB4 cells were cultured for 72 hours in the presence of benfotiamine (100 μM) and then subjected to Giemsa staining. Arrows indicate the presence of cytoplasmic vacuoles. (C) HL-60 cells were cultured in the presence of 0.25 μg/ml of cycloheximide (CHX) or tetrathylammonium (TEA) for 2 hours, cultured for an additional 12 hours with or without 100 μM benfotiamine (Benfo) and stained with Giemsa. A representative result of three independent experiments is shown. (D) Transmission electron microscopic images of HL-60 cells treated with (D.1, D.2) or without (D.3) benfotiamine. The characteristic signs of paraptosis in benfotiamine-treated cells with massive vacuolization (open arrowheads) are visible. The inset in D.1 is a higher magnification showing mitochondria enlargement. Fig. D.2 shows megavacuoles occupying the cytoplasm of benfotiamine-treated HL-60 cells (closed arrowheads). Fig. D.3 shows untreated HL-60 cells with normal mitochondria architecture and rough endoplasmic reticulum. Bar = 1μm.

To identify potential mechanisms mediating the antitumor properties of benfotiamine, we next performed mechanistic studies using primary blasts as the target and four leukemia cell lines that showed either resistance (TF-1 and THP-1 cells) or sensitivity (HL-60 and NB4 cells) to the antitumor effects of benfotiamine. Staining for annexin V showed that the treatment of leukemia cells with benfotiamine did not result in apoptosis, even after 96 hours of culture (**S1 Fig**). In addition, HL-60 cells treated with benfotiamine for up to 72 hours exhibited no morphological signs of differentiation, such as adhesion to plates or spreading (data not shown), and neither acquired CD14 of CD11c expression on their surfaces (**S2 Fig)**, indicating that the antiproliferative activity of benfotiamine is not associated with the induction of leukemia differentiation. Notably, Giemsa staining revealed that benfotiamine was able to induce cytoplasmic vacuolization in BM-S-blasts (**Fig 2A**) and HL-60 and NB4 cells (**Fig 2B)** but not in THP-1, TF-1 cells or PB-R-blasts (data not shown).

To identify the underlying processes causing the vacuolization in the benfotiamine-sensitive leukemia cells, we first investigated autophagy since this catabolic process is frequently associated with cytoplasmic vacuoles [19]. Benfotiamine-treated leukemia cells did not show signs of autophagy as demonstrated by the absence of LC3 protein, a marker of autophagy [19], in cellular extracts from benfotiamine-treated HL-60 cells (**S3 Fig)**. These results were further confirmed using a commercial assay that uses a fluorescent probe specific for autophagy (**S3 Fig)**. Notably, cytoplasmic vacuolization was attenuated when the benfotiamine-treated cells were pretreated with cycloheximide, indicating that the vacuole induction by benfotiamine requires new protein synthesis. In addition, vacuolization was prevented by tetrathylammonium, a specific BK channel inhibitor (**Fig 2C**). The fact that benfotiamine-induced vacuolization requires both new protein synthesis and a functional BK channel in the target cells suggest that the anti-leukemia activity of benfotiamine may be mediated through paraptosis, which is an alternative cell death mechanism recently described [20]. To further characterize the benfotiamine-induced vacuoles, transmission electron microscopy was performed in HL-60 cells exposed to benfotiamine. Compared to untreated cells, which showed normal cytoplasmic and nuclear structure (**Fig 2D.3**), HL-60 cells exposed to benfotiamine showed extensive cytoplasmic vacuolization and mitochondria enlargement (**Fig 2D.1**) with some cells having several megavacuoles occupying the cytoplasm (**Fig 2D.2**). Notably, despite the extensive mitochondria swelling, those cells showed no signs of nuclear fragmentation or chromatin condensation thus indicating that benfotiamine-induced cell death is consistent with paraptosis.

### Effects of benfotiamine on the activation of MAPK in leukemia cells

**Fig 3:**
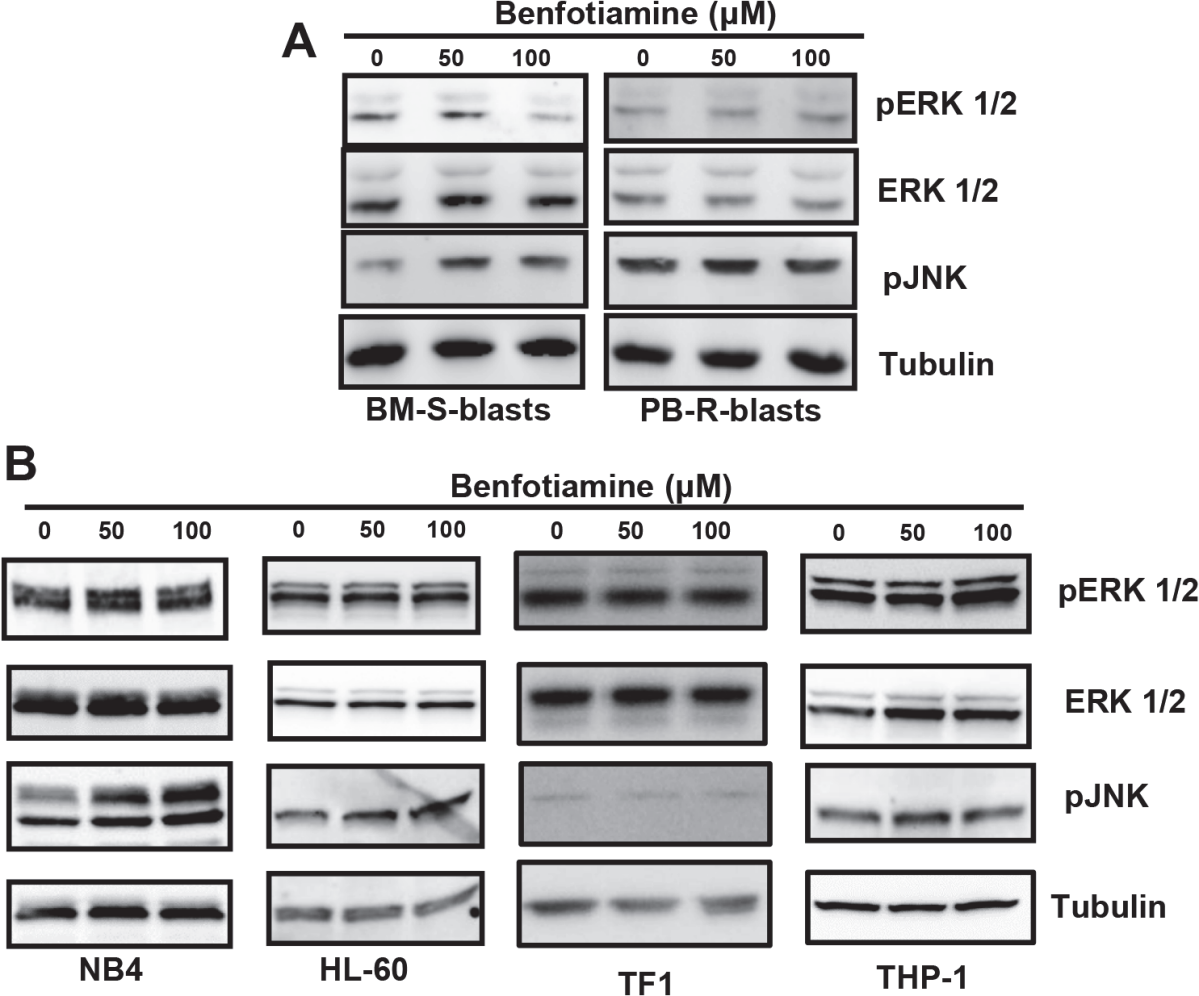
Modulation of MAPK members by benfotiamine. BM-S-blasts or PB-R-blasts (A) and HL-60, NB4, THP-1 and TF1 cells (B) were cultured for 24 hours with the indicated doses of benfotiamine and analyzed for phosphorylated ERK 1/2 and JNK1/2 via Western blotting. Representative results of three independent experiments are shown.

Next, the effects of benfotiamine on MAPKs, including ERK1/2, JNK1/2 and MAPK p38, were analyzed. The Western blotting analysis revealed that benfotiamine induced a dose-dependent inhibition of the ERK 1/2 activity and concomitantly enhanced the activation of JNK1/2 in the BM-S-blasts but not in the PB-R-blasts (**Fig 3A**).

Treatment of HL-60 and NB4 cells, which were found to be highly sensitive to the antiproliferative effects of benfotiamine, did not affect the constitutive phosphorylation of ERK 1/2, but rather resulted in the hyperactivation of JNK 1/2 kinase (**Fig 3B**). Conversely, benfotiamine did not affect the activities of MAPKs in TF-1 or THP-1 cells (**Fig 3B**). These results suggest that the antiproliferative effects of benfotiamine in leukemia cells involve the modulation of MAPK pathways.

### Effects of benfotiamine on cell cycle

**Fig 4:**
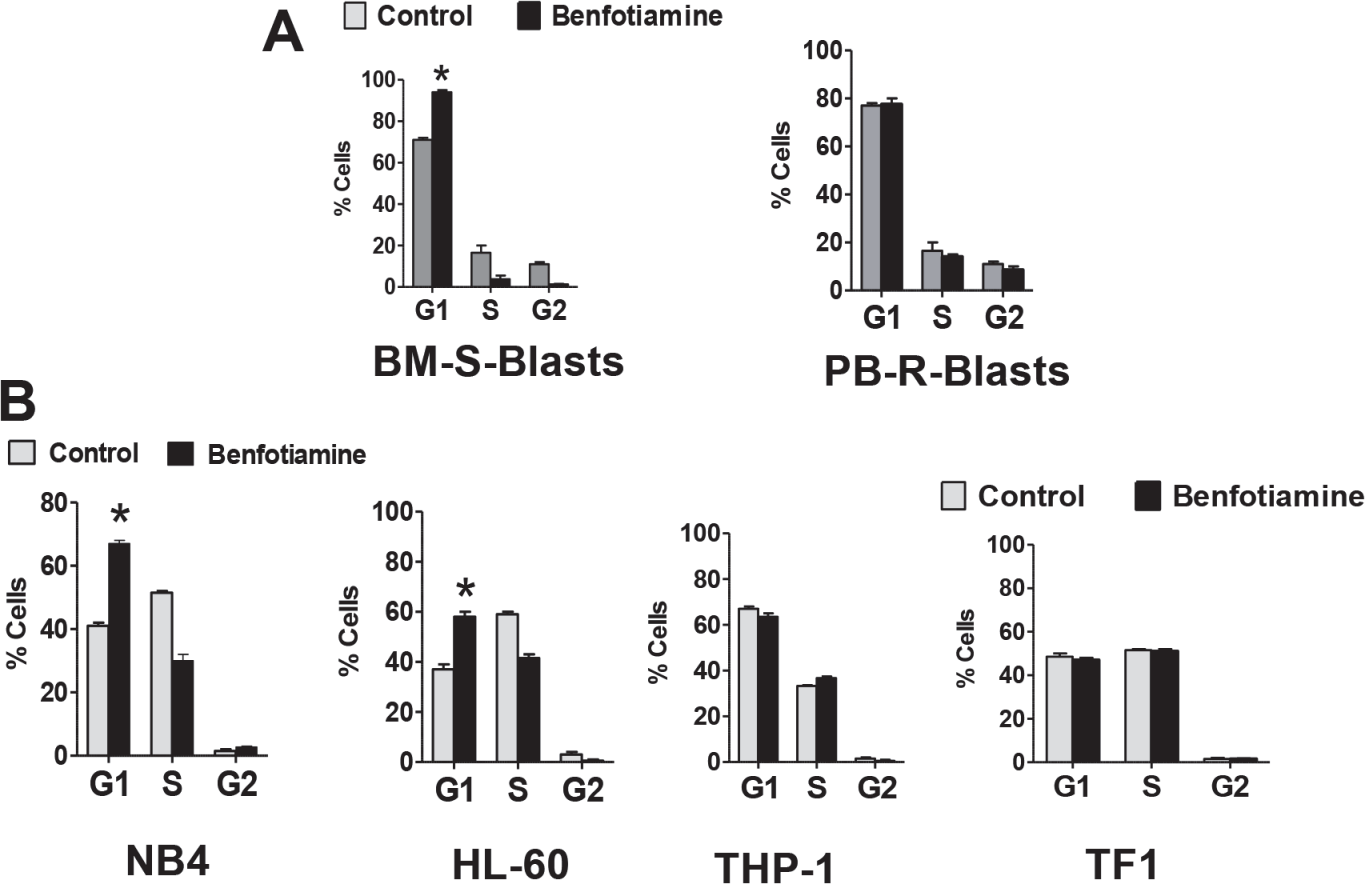
Effects of benfotiamine on cell cycle progression. BM-S-blasts and PB-R-blasts (A) or HL-60, NB4, THP1 and TF1 cells (B) were cultured for 24 hours in the presence or absence of benfotiamine (100 μM) and the percentages of cells in G_1_, S, and G_2_ phases were evaluated by flow cytometry. The figures indicate the means ± SD of summarized data from three independent experiments.

Next, we evaluated the effects of benfotiamine on the cell-cycle distribution of leukemia cells by flow cytometry. After 24 hours of treatment with benfotiamine, the percentage of BM-S-blasts, but PB-R-blasts, in the G_1_ phase increased significantly (**Fig 4A**).

G1 arrest with a concomitant reduction of cells in the S and G_2_–M phases was also observed in benfotiamine-treated HL-60 and NB4 cells but not in TF-1 or THP-1 cells (**Fig 4B**) suggesting a correlation between cell-cycle arrest and cell growth inhibition induced by benfotiamine.

### Modulation of cell-cycle and apoptosis-associated proteins by benfotiamine

**Fig 5:**
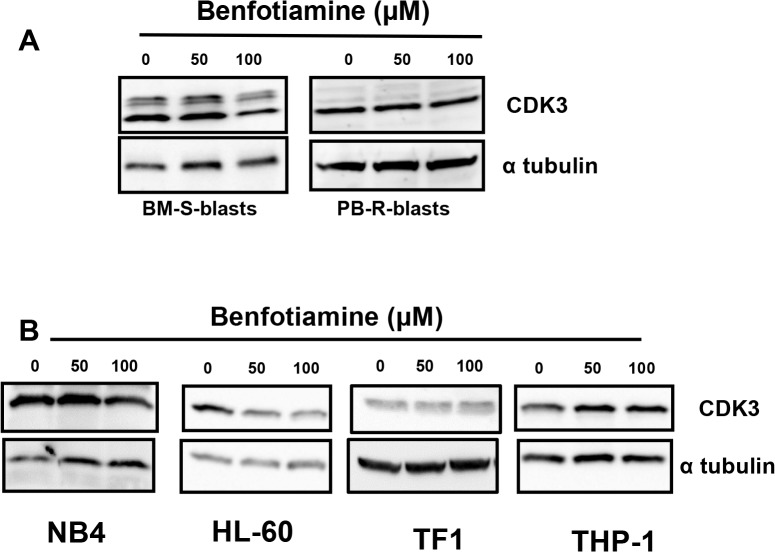
Modulation of CDK3 expression by benfotiamine. BM-S-blasts and PB-R-blasts (A) NB4, HL-60, THP-1 and TF1 cells (B) were cultured for 24 hours in the presence or absence of benfotiamine and the expression of CDK3 was analyzed via Western blotting. Representative results of three independent experiments are shown.

To clarify the molecular mechanisms underlying the benfotiamine-induced cell cycle arrest, the expressions of several cell cycle-related proteins were examined using Western blotting. Exposure to benfotiamine consistently decreased the expression of CDK3 in the BM-S-blasts but not in the PB-R-blasts (**Fig 5A**).

CDK3 inhibition was also observed in benfotiamine-treated HL-60 cells and NB4 cells. In contrast, benfotiamine did not affect the CDK3 expression in the resistant cell lines THP-1 and TF-1 (**Fig 5B**). The expressions of other cell cycle regulator proteins, including total and phosphorylated CDK2 and CDC2, were not altered in the benfotiamine-treated cells (**S4 Fig**). These findings indicate that a decrease in the level of CDK3 is a key event which mediates the antiproliferative activity of benfotiamine.

### Benfotiamine sensitizes AML cells to cytarabine-induced cytotoxicity

**Fig 6:**
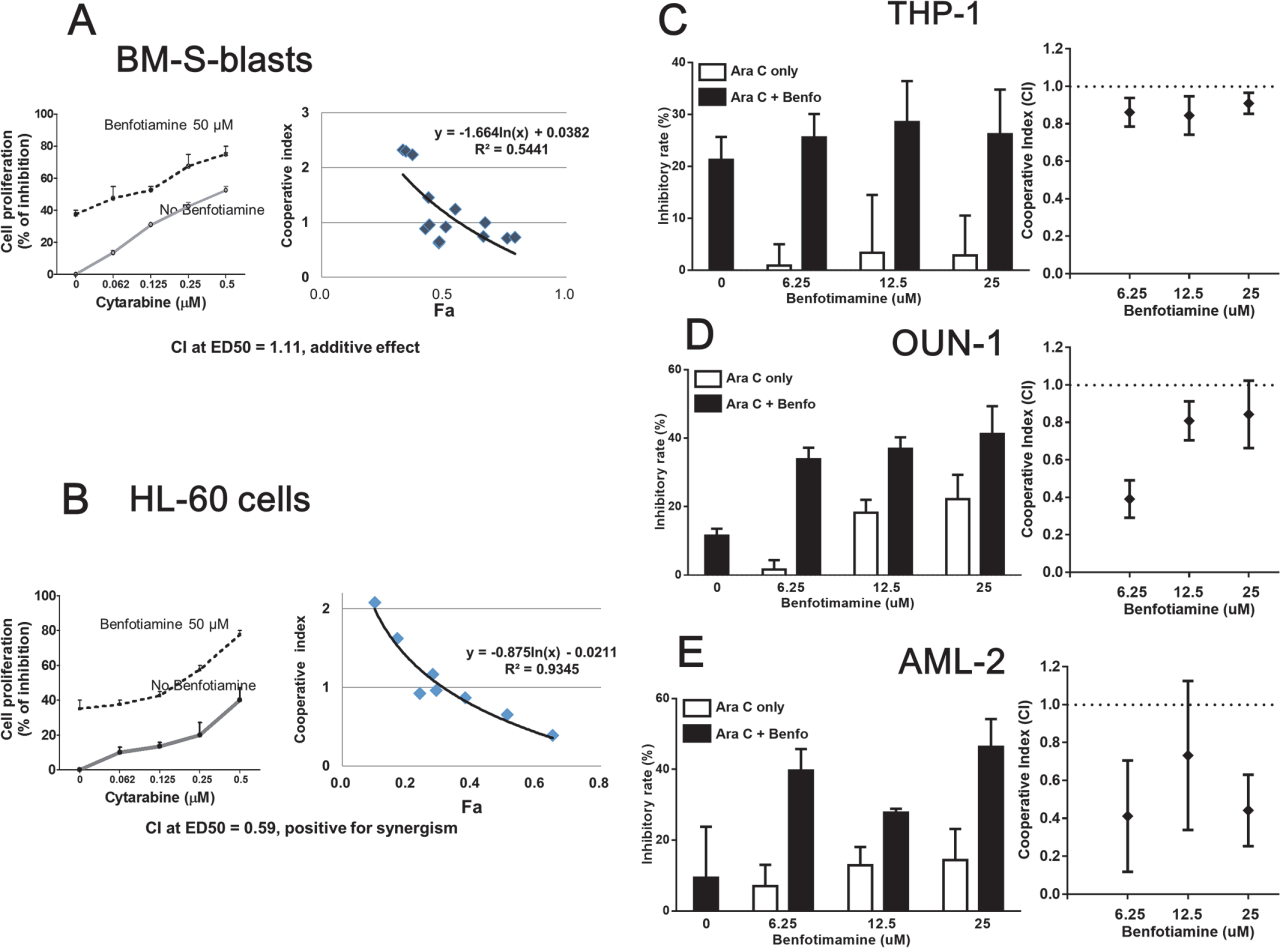
Benfotiamine acts synergistically with cytarabine to inhibit leukemia cell proliferation. BM-S blasts (A) and HL-60 cells (B) were cultured for 72 hours with various concentrations of cytarabine (Ara-C) alone or in combination with benfotiamine (50 μM) and the degree of cell viability was assessed using an MTT assay. THP-1 cells (C), OUN-1 cells (D) and AML-2 blasts (E) were cultured for 72 hours with Ara-C alone (THP-1, 10 nM; OUN-1 and AML-2, 50 nM) or in combination with the indicated doses of benfotiamine and the degree of cell viability was assessed using an MTT assay. The combination index (CI) was calculated to determine drug interactions using the CompuSyn software program.

When primary leukemia cells and leukemia cell lines, including HL-60, THP-1 and OUN1 cells, were treated with benfotiamine in combination with cytarabine (a cytotoxic drug commonly used to treat AML) *in vitro*, an increase in the antiproliferative activity of cytarabine was observed in all the cells tested. The combination index calculated using an isobologram analysis indicated that the growth inhibition of benfotiamine was additive against BM-S blasts (**Fig 6A)** and synergistic against HL-60 cells (**Fig 6B)**, as well as against THP-1, OUN1 cells and AML-2 primary leukemia cells **(Fig 6C–6E**).

Furthermore, in the combination studies benfotiamine was found to exert synergistic effects at lower doses (starting at 6 uM).

## Discussion

Extensive studies using *in vitro* and *in vivo* models have provided solid evidence of the beneficial effects of benfotiamine on glucose metabolism [6]. Therefore, this molecule is currently used to treat vascular complications associated with diabetes [21,22]. The present study showed for the first time the novel and unexpected finding that benfotiamine possess antitumor properties against leukemia cells.

Specifically, benfotiamine was shown to induce leukemia cell growth inhibition as a result of cell cycle arrest and paraptosis cells death.

Although the effects of benfotiamine on cell metabolism [6,23] may play a role in its antitumor activity, it is more likely that the antitumor effect is a unique property of this synthetic compound, presumably mediated by the compound’s benzoyl group, which shares some features with benzaldehyde, an organic compound with reported antitumor effects against several solid tumors [24,25]. Moreover, because benfotiamine is an S-acyl thiamine derivative, it has been suggested that the spontaneous rearrangement of S-acyl cysteine derivatives into N-acyl cysteine derivatives may occur *in vivo* [26]. Interestingly, N-acyl cysteine derivatives have been reported to interfere with pyrimidine synthesis and related signaling, leading to leukemia cell growth inhibition [27]. The extreme sensitivity of HL-60 cells to benfotiamine observed in our study may support this hypothesis, as earlier *in vitro* studies documented that this cell line is particularly sensitive to the inhibitory effects of N-acyl cysteine derivatives [27].

One key finding of this study was the identification of cytoplasmic vacuolization induced by benfotiamine in the benfotiamine-sensitive leukemic cells. The absence autophagy, necrosis or apoptosis, together with the fact that vacuoles induced by benfotiamine was prevented by a BK channel inhibitor and by cycloheximide, indicates that benfotiamine most likely induces paraptosis cell death. Paraptosis is a recently described programmed cell death pathway characterized by cytoplasmic vacuoles that does not involve caspase activation or morphologic signs of apoptosis [20]. Although the exact mechanisms triggering paraptosis have not been completely elucidated, it is known that paraptosis is associated with the disruption of internal potassium ion homeostasis due to the opening of BK channels [28,29] and is dependent on new protein synthesis [20,30]. A previous report documented that IGFIR-induced paraptosis requires the activation of both the MAPK/ERK and JNK pathways [30]. Consistent with these studies, we also found MAPK modulation in leukemia cells treated with benfotiamine and these events appear to be associated with the induction of paraptosis. However, it must be noted that although we found that benfotiamine induces JNK1/2 activation in HL-60 and NB4 cells and BM-S blasts, contrary to the report by Sperandio and colleagues, benfotiamine treatment inhibited the MAPK/ERK pathway in BM-S blasts but did not affect the activation status of that pathway in HL-60 or NB4 cells; therefore, in our system, only JNK1/2 activation was shown to be critical for paraptosis induction by benfotiamine. Differences in the paraptosis inducer (IGFIR vs. benfotiamine) and different cellular systems (HEK cells vs. leukemia cells) may account for these apparent discrepancies between our findings and the previous study [30].

There are some limitations associated with this study. Although we have identified the cellular events that are associated with paraptosis cell death induced by benfotiamine in malignant cells, the factors that determine cell sensitivity or resistance to this compound remain to be determined. We hypothesize that, at least during *in vitro* condition; cell sensitivity to benfotiamine is influenced by the amount of intact benfotiamine that reaches the intracellular compartment of cultured cells. In this regard, it has been reported that the capacity of intact benfotiamine to cross the cell membrane is very poor [31] and therefore dephosphorylation of benfotiamine to the cell permeable form S-benzoylthiamine is an event required for the diffusion of benfotiamine across the cell membrane [26,31]. Benfotiamine dephosphorylation appears to be controlled by the expression of ecto-alkaline phosphatases in the cultured cells [31]. Although highly speculative, it is plausible that cells expressing higher levels of ecto-alkaline phosphatases may be more sensitive to the antitumor effects of benfotiamine. Interestingly HL60 cells, which showed a high sensitivity to benfotiamine in the current study, have been reported to express functional ecto-alkaline phosphatases [32]. Alternatively, cell sensitivity to the antitumor effects of benfotiamine may be determined by the availability of reduced folate carrier-1 (RFC-1) on the cell surface, since previous reports have shown that a small amount of intact benfotiamine can enter the cells via this transport system [26]. Although we did not determine the expression levels of RFC-1 on leukemia cells in the present study, it can be speculated that cells expressing high levels of RFC-1 are subjected to higher levels of intact benfotiamine intracellularly and thus cells become more sensitive to the antitumor effects of this compound. Further studies using proteomic approaches, gene expression arrays or similar screening strategies will be very useful to identify potential cellular signatures that may predict the leukemia cell sensitivity to benfotiamine.

Despite being discovered more than 40 years ago, the pharmacokinetic data of benfotiamine in humans is limited [31,33]. Benfotiamine has been considered to be a pro-drug that, following oral administration, is dephosphorylated in the gut to benzoylthiamine and then transformed into thiamine. Therefore it has been assumed that the exposure of cells *in vivo* to intact benfotiamine is minimal. Ziems and colleagues [33] reported that after oral administration of 250 mg of benfotiamine in healthy volunteers, a small amount of intact benfotiamine (peak plasma concentration, 10 nM) enters the blood circulation and can be distributed into body cells. In the present study, the antitumor effects of benfotiamine on leukemia cells were observed at high concentrations that may be difficult to achieve *in vivo*; however, in our study, antitumor effects were observed at concentrations of benfotiamine that are actually lower than those used in other cellular systems with no cellular toxicity reported. For example, in previous studies, 200 uM of benfotiamine induced beneficial effects on glucose metabolism but not toxicity in cultured progenitor endothelial cells [34] or in primary human myotubes [26].

It is unknown whether high doses of oral benfotiamine result in a higher absorption of intact benfotiamine in humans. Recent clinical trials reported that benfotiamine at very high doses (900 mg/day) was well tolerated [21,22], however, the safety of even higher doses of benfotiamine remains to be determined. We envisage that the antitumor potential of benfotiamine could be improved by enhancing the absorption of intact benfotiamine to increase the amount of benfotiamine that reaches the blood circulation system. This strategy could be achieved by introducing minor modifications in the benfotiamine molecule, preventing the transformation of benfotiamine into thiamine.

Benfotiamine is a clinically promising agent for AML, especially in patients who are not eligible for standard chemotherapy. Moreover, the observation that benfotiamine synergizes with cytarabine to eliminate myeloid leukemia cells *in vitro* suggests that benfotiamine, in combination with currently available chemotherapeutic protocols, is an attractive therapeutic approach that should be further investigated in clinical trials.

## Supporting Information

S1 Table Patient characteristics. (PDF)

S1 Fig Benfotiamine does not induce apoptosis in leukemia cells. Leukemia cells were cultured for the indicate times with or without benfotiamine and stained with annexin V and 7AAD to assess apoptosis.
(TIF)

S2 Fig Benfotiamine and the differentiation of HL-60 cells. CD14 and CD11c expression on HL-60 cells treated with benfotiamine for 72 hours
(TIF)

S3 Fig Benfotiamine and autophagy in HL-60 cells. (A) Leukemia cells were treated with benfotiamine for the indicated times and the expression of LC3 protein was assessed by Western blotting. (B) Cells were treated for 24 h and stained with an autophagy kit and assessed by Flow Cytometry.
(TIF)

S4 Fig Benfotiamine and cell cycle proteins in HL-60 and THP-1 cells. HL-60 and THP-1 cells were cultured for 24 hours with the indicated dose of benfotiamine and the expression of cell cycle regulator proteins was analyzed by Western blotting.
(TIF)
