# Supplementary material for: Paraptosis Cell Death Induction by the Thiamine Analog Benfotiamine in Leukemia Cells
Source: PLoS One. 2015 Apr 7;10(4):e0120709. doi: 10.1371/journal.pone.0120709 (PMC4388699; doi:10.1371/journal.pone.0120709)
Supplement: S1 Table — (PDF) [file pone.0120709.s001.pdf]

Table S1. Patient characteristics

Sample No	Gender	Age	AML stage (diagnosisWHO//FAB)	Cytogenetic
AML-1	M	66	AML/MRC//M5a	46,X,-Y,+mar1 [17/20], 46, XY[3/20],
AML-2	F	63	AML:t(9;11)(p22;q23)//M5a	47,XX,+8,t(9;11)(p22;q23) [7/20], 47, idem,t(4;5)(p16;q31)[2/20],52,idem, +2,+6,+19,+21,+22[11/20]
AML-3	F	38	AML NOS//M2 onset	46,XX,-9,-16,+2mar [1/20], 46, XX[19/20],
AML-4	M	53	AML NOS//M1	46,XY [20/20]
AML5	F	53	AML NOS// M4 3rd relapse post UR BMT, post CBT	complex [7/20], 46, XX[13/20],
AML-6	M	39	t-MN//AML (Post B-ALL)	47,XY,dup(1)(q32q12),+8 [20/20]

AML/MRC: Acute Myeloid Leukemia with myelodysplasia-related changes

AML NOS: Acute myeloid leukemia, not otherwise specified

t-MN: Therapy-related myeloid Neoplasia
