# Supplementary figures and images for: Paraptosis Cell Death Induction by the Thiamine Analog Benfotiamine in Leukemia Cells
Source: PLoS One. 2015 Apr 7;10(4):e0120709. doi: 10.1371/journal.pone.0120709 (PMC4388699; doi:10.1371/journal.pone.0120709)

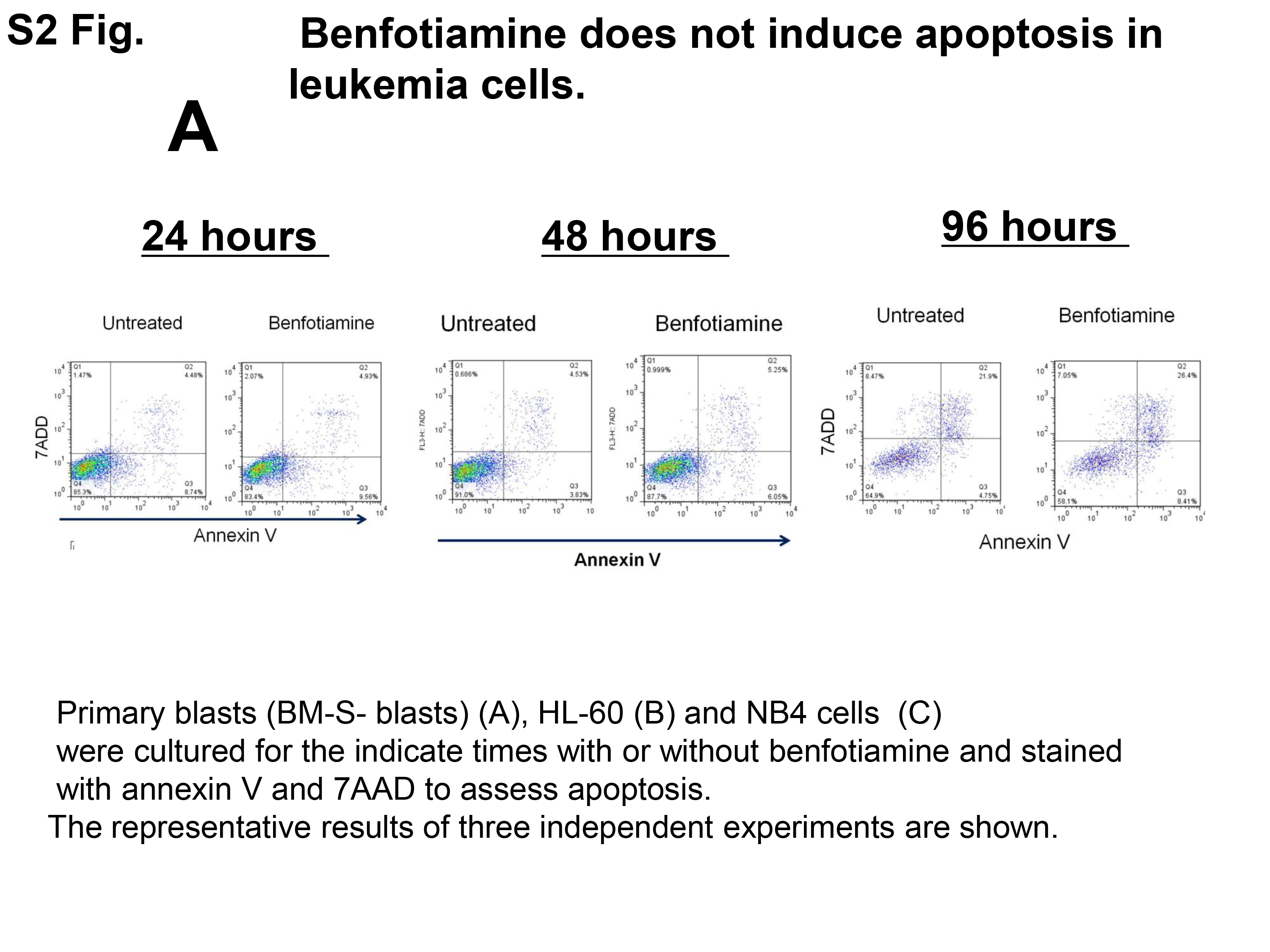

Supplement: S1 Fig — Leukemia cells were cultured for the indicate times with or without benfotiamine and stained with annexin V and 7AAD to assess apoptosis. (TIF) [file pone.0120709.s002.tif]

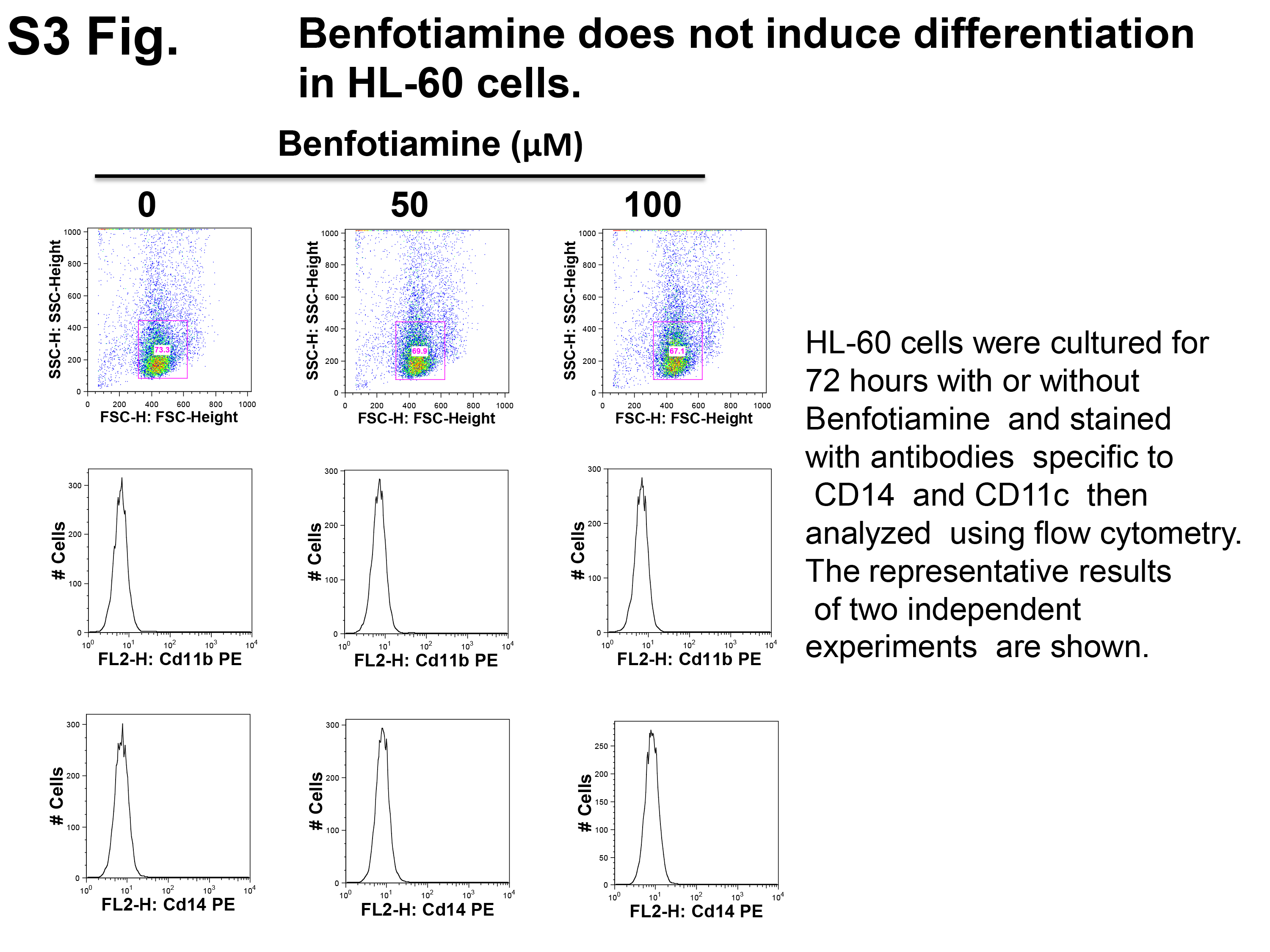

Supplement: S2 Fig — CD14 and CD11c expression on HL-60 cells treated with benfotiamine for 72 hours (TIF) [file pone.0120709.s003.tif]

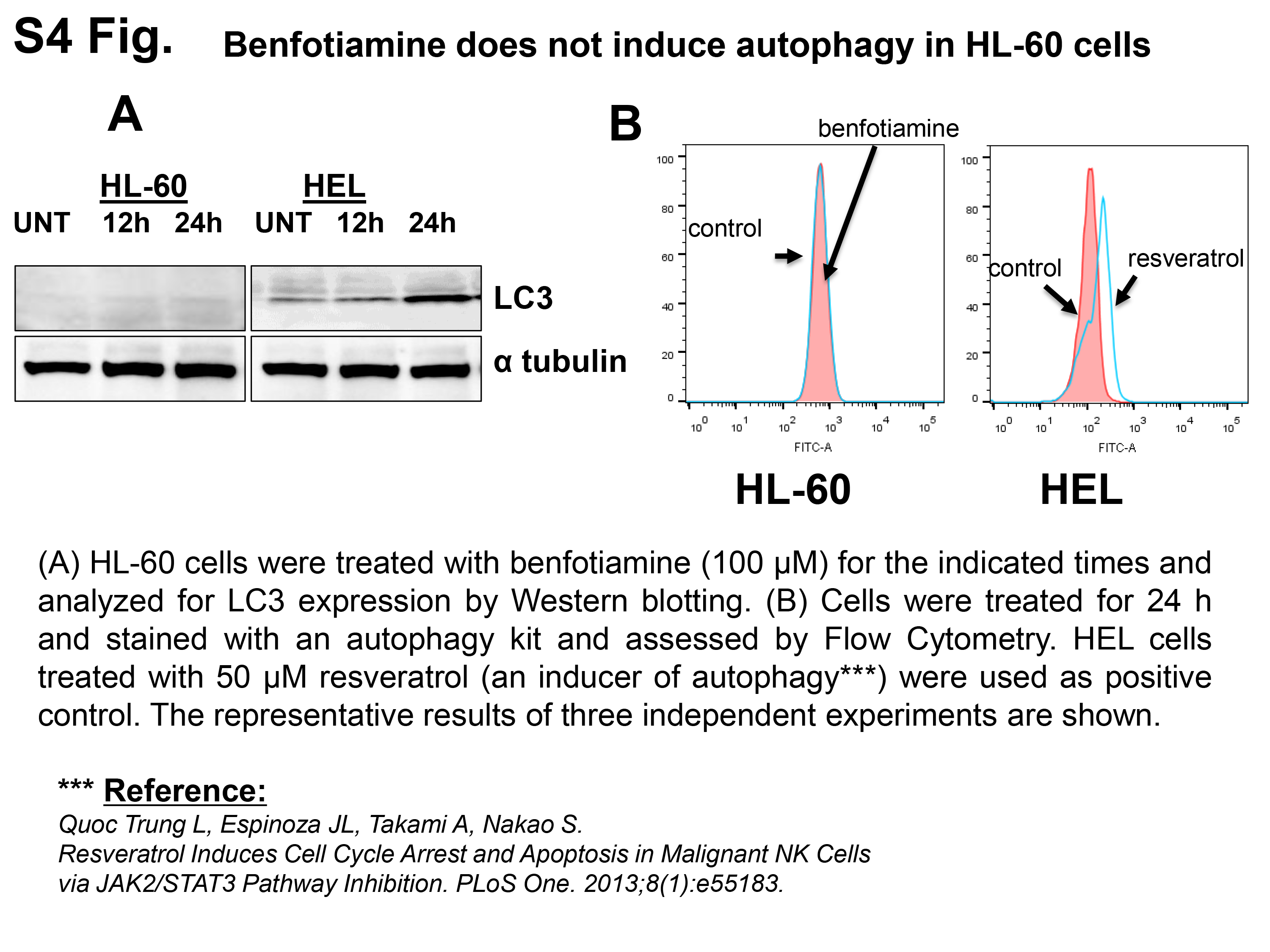

Supplement: S3 Fig — (A) Leukemia cells were treated with benfotiamine for the indicated times and the expression of LC3 protein was assessed by Western blotting. (B) Cells were treated for 24 h and stained with an autophagy kit and assessed by Flow Cytometry. (TIF) [file pone.0120709.s004.tif]

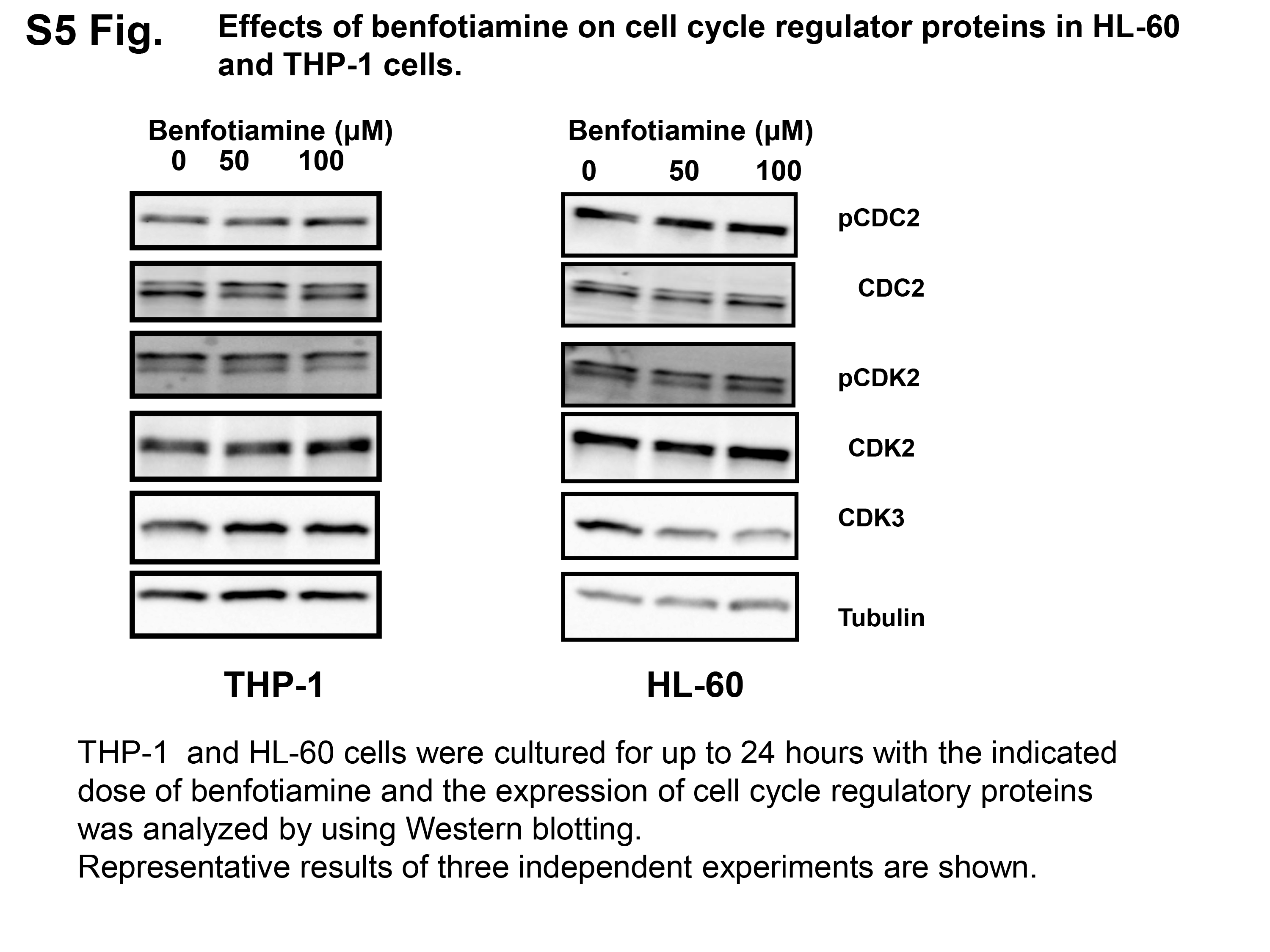

Supplement: S4 Fig — HL-60 and THP-1 cells were cultured for 24 hours with the indicated dose of benfotiamine and the expression of cell cycle regulator proteins was analyzed by Western blotting. (TIF) [file pone.0120709.s005.tif]
